# Genetically influenced tobacco and alcohol use behaviors impact erythroid trait variation

**DOI:** 10.1371/journal.pone.0309608

**Published:** 2024-09-05

**Authors:** Shriya Shivakumar, Madison B. Wilken, Victor Tsao, Bárbara D. Bitarello, Christopher S. Thom

**Affiliations:** 1 Division of Neonatology, Children’s Hospital of Philadelphia, Philadelphia, PA, United States of America; 2 Biology Department, Bryn Mawr College, Bryn Mawr, PA, United States of America; 3 Department of Pediatrics, University of Pennsylvania Perelman School of Medicine, Philadelphia, PA, United States of America; Imam Abdulrahman Bin Faisal University, SAUDI ARABIA

## Abstract

Genome wide association studies (GWAS) have associated thousands of loci with quantitative human blood trait variation. Loci and related genes that impact blood trait variation may regulate blood cell-intrinsic biological processes, or alternatively impact blood cell development and function via systemic factors. Clinical observations have linked tobacco or alcohol use with altered blood traits, but these trait relationships have not been systematically explored at the genetic level. Applying a Mendelian randomization (MR) framework to GWAS summary statistics, we explore relationships between smoking and drinking behaviors with 15 quantitative blood traits. We find that the effects of smoking and drinking are confined to red blood cell traits. An instrumental variable (IV) comprised of 113 single nucleotide polymorphisms (SNPs) associated with smoking initiation is associated with decreased hemoglobin (HGB: Effect = -0.07 standard deviation units [95% confidence interval = -0.03 to -0.10 SD units], P = 1x10^-4^), hematocrit (HCT: Effect = -0.06 [-0.03 - -0.09] SD units, P = 4x10^-4^), and red blood cell count (RBC: Effect = -0.05 [-0.02 - -0.09] SD units, P = 5x10^-3^) without impacting platelet count (P = 0.9) or white blood cell count (P = 0.6). Similarly, an IV associated with an increased number of alcoholic drinks consumed per week is associated with decreased HGB (Effect = -0.22 [-0.42 - -0.02] SD units, P = 3x10^-2^) and RBC (Effect = -0.27 [-0.51 - -0.03] SD units, P = 3x10^-2^). Using multivariable MR and causal mediation analyses, we find that an increased genetic predisposition to smoking initiation is associated with increased alcohol intake, and that alcohol use mediates the genetic effect of smoking initiation on red blood cell traits. These findings demonstrate a novel role for genetically influenced behaviors on human blood traits, revealing opportunities to dissect related pathways and mechanisms that influence hematopoiesis and blood cell biology.

## Introduction

Tobacco and alcohol use are prevalent behaviors with detrimental effects on human health. More than 85% of U.S. adults over the age of 18 report alcohol use and >12% (>30 million) actively smoke cigarettes [1,2]. Cardiovascular disease risks associated with alcohol [3] and tobacco [4] use are well documented, including increased risks of coronary artery disease (CAD), hypertension, atrial fibrillation, coronary thrombosis and heart failure [5,6]. In contrast, the effects of cigarette smoking and alcohol consumption on other organ systems–e.g., blood traits–are less well understood.

Blood cells are responsible for oxygen transport, hemostasis, and immune function, as well as physiologic responses to systemic stress, vascular and endothelial functions, and toxin clearance [7]. Variations in blood traits can reflect altered blood cell development and/or differences in blood cell functions, including how long cells remain in circulation before being cleared [8]. Clinical observational studies have linked tobacco and alcohol use with blood trait variation, such as altered hemoglobin levels and red blood cell counts [9–13]. However, some findings are contradictory with regards to the direction of effect for tobacco use on these blood cell parameters.

Tobacco smoking has been linked with anemia (low hemoglobin levels) from blood loss or suppression of erythropoiesis (red blood cell production) [14]. Paradoxically, multiple studies have also associated smoking with higher hemoglobin levels [9,12]. Higher hemoglobin levels have been presumed to be due to a compensatory hypoxic response to chronic carbon monoxide exposure caused by smoking, resulting in increased red blood cell production. Alcohol use has generally been associated with anemia via effects on erythrocyte production, metabolism, and function [13]. A limitation to interpreting these observations is that they could reflect several cause-and-effect explanations (e.g., direct causation, confounding, or reverse causation), which can be difficult to parse or measure in a clinical context.

Thus, outstanding questions are i) how tobacco and alcohol use affect blood cell traits and ii) whether these associations are directly causal or a consequence of shared confounders. These are important questions, given that variation in blood traits can impact laboratory test inferences and clinical management. We also wanted to define how tobacco and alcohol use influence blood cell traits at a genetic level to better understand how these systemic exposures impact blood development and blood disease phenotypes. Factors that impact blood cell development and function have broad implications for human health.

We hypothesized that genetic methods might clarify how tobacco and alcohol use behaviors impact blood cell trait variation. Well-powered genome wide association studies (GWAS) have linked thousands of genomic loci with altered quantitative blood traits—including variations in the size and count of red blood cells, white blood cells, and platelets—in otherwise healthy individuals [15–17]. GWAS have also identified loci that influence tobacco and alcohol use [18]. These loci impact how likely it is for someone to initiate smoking or drink alcohol more frequently (number of drinks per week), among other self-reported behaviors [18]. Interestingly, there was a high genetic correlation (*r_g_*~0.34) between drinks per week (DrnkWk) and smoking initiation (SmkInit) [18]. These well-powered blood trait and substance use GWAS provide an opportunity to analyze the genetic influence of smoking and alcohol use behaviors on blood trait variation, with increased power to discover associations as compared with a prior study that linked smoking to altered blood traits based on a restricted number of genomic loci [19].

Here, we use a Mendelian randomization (MR) framework to assess causal effects of tobacco and alcohol use on 15 quantitative blood traits. We focus on GWAS summary statistics from individuals with European ancestry. MR leverages the random allocation of single nucleotide polymorphisms (SNPs) at meiosis to make causal inferences about the effects of a genetically influenced exposure trait on an outcome phenotype, provided certain conditions are met [20]. Multivariable MR (MVMR) extends this method by jointly estimating the effects of multiple exposures on an outcome [21]. In this study, we use MR methods to examine the effects of genetically predicted alcohol or tobacco use (exposures) on quantitative blood trait variation (outcomes). Genetically predicted increases in tobacco or alcohol use decreased hemoglobin levels and red blood cell counts, with minimal effects on platelet or white blood cell traits, clarifying effects of these behaviors on red cell indices at a population level.

## Results

### Increased genetically predicted risk of smoking initiation decreases hemoglobin, hematocrit, and red blood cell count

**Fig 1:**
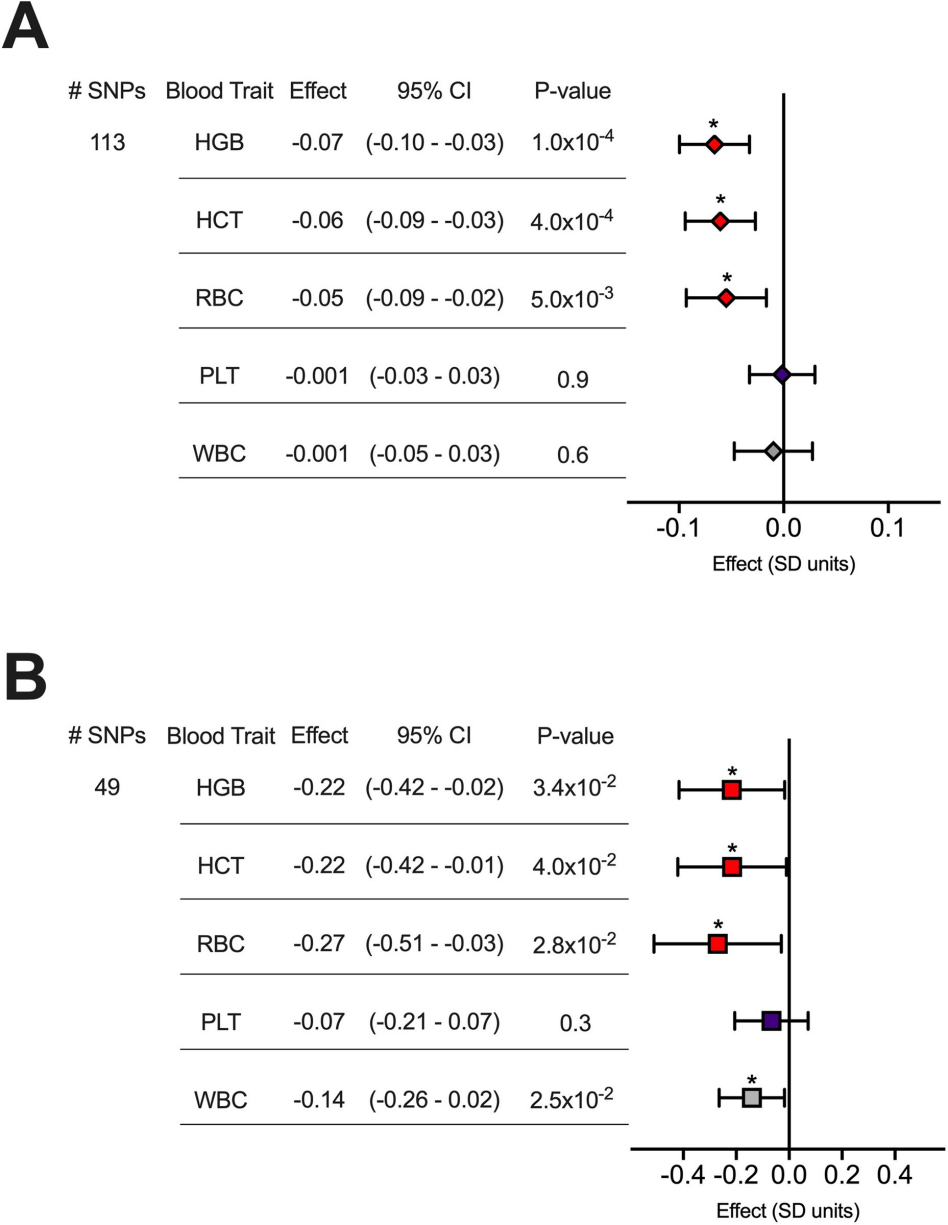
Two sample MR effect estimates for smoking initiation (SmkInit) or alcohol use (DrnkWk) on blood traits. (**A-B**) MR results using the inverse variance weighted method on five blood traits. Values for effect sizes on the indicated blood traits reflect (**A**) a 2-fold increase in SmkInit risk or (**B**) a 1 SD unit increase in alcoholic drinks per week. HGB, hemoglobin, HCT, hematocrit, RBC, red blood cell, PLT, platelet count, WBC, white blood cell count. Red indicates erythroid traits. Bars indicate 95% confidence intervals. *p<0.05.

We conducted two sample MR experiments to assess whether genetically determined smoking initiation (SmkInit) risk impacted one or more of 15 quantitative blood traits (**S1 Table**). Definitions for these blood traits, as well as other genetic traits used in this study, can be found in **S1 Table**. We first constructed an instrumental variable (IV) for SmkInit comprising 113 linkage-independent SNPs. By MR, this IV for SmkInit was negatively associated with quantitative measures of hemoglobin (HGB: Effect = -0.07 standard deviation units [95% CI: -0.03 to -0.10 SD units], P = 1x10^-4^), hematocrit (HCT: Effect = -0.06 [-0.03 - -0.09] SD units, P = 4x10^-4^), and red blood cell count (RBC: Effect = -0.05 [-0.02 - -0.09] SD units, P = 5x10^-3^) by the inverse variance weighted (IVW) method (**Fig 1A**). The effects of SmkInit did not extend to quantitative platelet (P = 0.9) or white blood cell (P = 0.6) traits (**Figs 1A** and **S1**).

To ensure that these results were valid and robust, we conducted tests for weak instrument bias, sensitivity, reverse causality, and horizontal pleiotropy. The IVs used for these experiments, and all other experiments in this manuscript, were not subject to weak instrument bias (F statistic > 10 in all cases, **S2 Table**) [22]. Sensitivity analyses using weighted median and MR Egger methods showed directionally consistent results for smoking initiation on blood traits (**S2 Fig**). We found non-significant MR Egger intercept values (all p>0.05), arguing against horizontal pleiotropy. Reverse causality experiments also supported a directional effect from SmkInit on red blood cell (“erythroid”) traits, including HGB, HCT, and RBC (**S3 Fig**). Overall, these results show that an increased genetically influenced risk of smoking initiation is associated with decreased HGB, HCT and RBC.

UK BioBank (UKBB) participants comprised significant percentages of both exposure and outcome GWAS [15,16,18]. Although MR results often remain valid despite sample overlap in GWAS summary statistics from large consortia [23], we wanted to estimate effects of smoking in the absence of sample overlap. When we performed two sample MR using a SmkInit IV from data that excluded UKBB participants (n = 50 SNPs), we identified negative effects on hemoglobin (Effect = -0.10 [-0.20 - -0.0004] SD units, P = 4.9x10^-2^) and red blood cell count (Effect = -0.13 [-0.25 - -0.01] SD units, P = 4x10^-2^) (**S4 Fig**).

Next, we used two sample MR to analyze the effects of other genetically determined smoking traits–smoking cessation (SmkCess), smoking heaviness as quantified by cigarettes smoked per day (CigPerDay), and lifetime smoking score (LfSmk, a measure of the duration and heaviness of the smoking habit [24,25])–on blood trait variation. We found no significant effects of SmkCess (RBC Effect = 0.03 [-0.041 to 0.10] SD units, P = 0.4), CigPerDay (RBC Effect = -0.05 [-0.10–0.006] SD units, P = 0.08), or LfSmk (RBC Effect = -0.04 [-0.12–0.05] SD units, P = 0.4) on erythroid traits (**S5 Fig**). We note that the IVs for SmkCess (n = 10 SNPs), CigPerDay (n = 30 SNPs), and/or LfSmk (n = 159 SNPs) may have been underpowered to detect significant associations, despite adequate F statistics (**S2 Table**). Clinical observations have proposed that increased hemoglobin and red cell counts among chronic tobacco smokers may represent a compensatory response to low-level carbon monoxide poisoning, which decreases the oxygen-carrying capacity of the blood [9,12]. However, our experiments revealed a null effect for LfSmk on red cell traits, arguing against a genetically determined link between chronic smoking and higher hemoglobin levels. Instead, unmeasured confounders may be responsible for clinically observed links between chronic tobacco smoking and higher hemoglobin.

### Genetically predicted body mass index does not mediate the effects of SmkInit on erythroid traits

Having confirmed that SmkInit IVs are negatively associated with three genetically predicted erythroid traits, we assessed whether this was a direct effect of smoking or if other phenotypes mediated this effect. Our prior work identified body mass index (BMI) as a genetically correlated trait to SmkInit that mediated effects of SmkInit on type 2 diabetes (T2D) risk [25]. We also previously showed that genetically predicted BMI negatively impacts genetically predicted blood cell traits [26]. These latter effects of BMI on blood traits were similar to the effects of SmkInit. Thus, we tested if BMI or other cardiometabolic/cardiovascular traits mediated the effects of SmkInit on blood traits.

We used two sample MR to ascertain whether a range of anthropometric, metabolic, and cardiovascular traits had any genetic impact on blood trait variation. Of the traits investigated, only BMI had a negative effect on blood traits that was akin to SmkInit (**S6 Fig**). Unlike SmkInit, however, the effects of BMI on blood traits extended across blood lineages including negative effects on red blood cell, white blood cell, and platelet traits (**S6 Fig**) [26].

We also found that the negative effects of BMI on quantitative blood traits were counterbalanced by positive effects of WHR (waist-hip ratio) on blood traits, as previously shown [26]. WHR had positive effects on red blood cell, white blood cell, and platelet traits (**S6 Fig**). We also noted a positive effect of depression on blood traits across lineages (**S6 Fig**). In summary, these findings suggested that BMI was the only potentially relevant mediating trait for SmkInit, since both BMI and SmkInit impacted blood traits in a similar negative direction.

We then used MVMR to test if BMI mediated the effects of SmkInit on erythroid traits. After adjusting SmkInit effects for BMI, SmkInit retained consistent effect sizes and significance estimates, with some loss of power (**S7 Fig**). Residual effects of SmkInit after adjustment included negative effects on hemoglobin (HGB: Effect = -0.05 [-0.02 - -0.09] SD units, P = 5x10^-3^), hematocrit (HCT: Effect = -0.05 [-0.01 - -0.08] SD units, P = 1x10^-2^), and red blood cell count (RBC: Effect = -0.04 [-0.08–0.001] SD units, P = 0.06) that were directionally consistent with our two sample MR findings. These results argued against BMI as a mediator for the effects of SmkInit on erythroid traits.

### Genetically predicted alcohol consumption mediates the effects of SmkInit on erythroid traits

We next considered genetically predicted alcohol consumption as a phenotype that could potentially mediate the effects of SmkInit on blood trait variation, given clinical links between increased use of alcohol and tobacco [27] and strong genetic associations between SmkInit and the number of alcoholic drinks consumed per week (DrnkWk, r_g_ = 0.34, p = 7x10^-63^ [18]).

Like SmkInit, an increase in DrnkWk negatively impacted HGB (Effect = -0.22 [-0.42 - -0.02] SD units, P = 3x10^-2^), HCT (Effect = -0.22 [-0.42 - -0.01] SD units, P = 4x10^-2^), and RBC (Effect = -0.27 [-0.51 - -0.03] SD units, P = 3x10^-2^) by the IVW method (**Fig 1B**). These directional results were consistent across other MR methods (**S2** and **S8 Figs**), without evidence of horizontal pleiotropy (p>0.05 for all MR Egger intercepts). These findings aligned with clinical observations related to the hematologic effects of alcoholism, including increased anemia risk [13].

**Fig 2:**
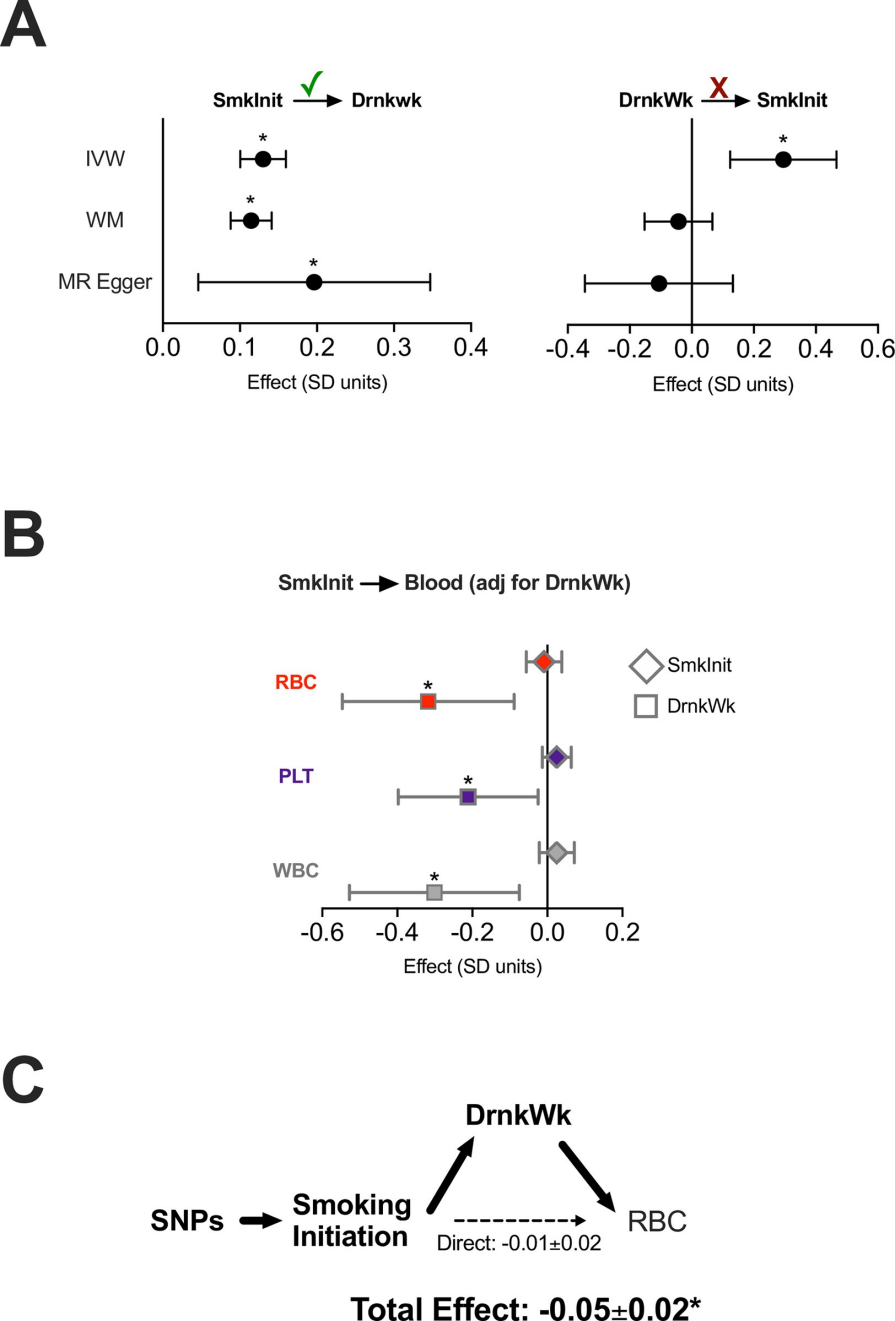
Genetically predicted alcohol use mediates the genetic effect of smoking initiation on erythroid traits. (**A**) By MR, an increased risk of SmkInit increases Drinks per week (DrnkWk) by inverse variance weighted (IVW), weighted media (WM), and MR Egger methods. However, increased genetically determined Drinks per week does not consistently increase SmkInit risk across MR methods. Check mark reflects MR Steiger ‘correct causal estimate’ relationship. (**B**) By MVMR, using an instrumental variable for SmkInit adjusted for Drinks per week, the effects of SmkInit on quantitative blood traits were nullified. Effects of Drinks per week on blood traits remain significantly negative. Bars indicate 95% confidence intervals. (**C**) Mediation analysis showed that SmkInit made a statistically insignificant direct effect on RBC, whereas the total effect (including indirect effects through DrnkWk) negatively impacts RBC. *p<0.05.

We then investigated whether SmkInit and DrnkWk impacted erythroid traits through common genetic mechanisms. We first confirmed by MR that an increased SmkInit risk portended an increase in DrnkWk (**Fig 2A**). Reverse causality and MR Steiger tests supported a unidirectional effect of increased SmkInit on increased DrnkWk (MR Steiger p = 2x10^-67^, sensitivity ratio 3.1, **Fig 2A**). This confirmed a genetic interaction between SmkInit and DrnkWk, and furthermore argued that an increase in genetically influenced SmkInit increases DrnkWk, but not vice versa.

Given this genetic interaction, we next used MVMR to determine if DrnkWk mediated the effects of SmkInit on blood traits. By MVMR, adjustment for DrnkWk abrogated the effects of SmkInit on erythroid traits (**Figs 2B** and **S9**). This indicates that DrnkWk was responsible for essentially all the effects of SmkInit on these traits. We further confirmed this intuition by formal mediation experiments, which revealed that SmkInit impacted RBC through effects mediated by DrnkWk (**Fig 2C**). Thus, a genetic predisposition for increased alcohol consumption mediates the effects of SmkInit on RBC.

We also used MVMR experiments to assess if chronic tobacco use, as measured by LfSmk, affected erythroid traits via DrnkWk. These findings were less clear. The effects of LfSmk on erythroid traits were more positive after adjustment for DrnkWk, but statistically non-significant (P = 0.2–0.8 for RBC, HGB, and HCT) (**S10 Fig**). Multivariable results with LfSmk did reveal a significant increase in WBC (Effect = 0.15 [0.04–0.26] SD units, P = 9x10^-3^) (**S10 Fig**), in alignment with clinical and genetic studies linking chronic tobacco use with increased inflammation and WBC count [10,19].

## Discussion

Systemic factors and disease states, including cardiovascular disease [28,29] and obesity [26], can impact blood cell formation and function. The MR framework is a good way to ascertain causal genetic interactions, given its independence from confounding effects that can bias observational studies [20]. Here, we used MR to reveal causal effects of genetically determined tobacco smoking and alcohol drinking on blood traits, providing novel insights that in some cases ran counter to clinical observations.

A genetic predisposition to initiate smoking (SmkInit) decreases red blood cell count, hemoglobin, and hematocrit (**Fig 1A**). This is primarily due to a concomitant increase in alcohol use (DrnkWk), which itself has a negative effect on these red cell traits (**Fig 1B**). Other potential confounders for SmkInit were not responsible for these effects (**S6 Fig**). Over the long term, the negative effects of smoking initiation on red cell traits are nullified by increased tobacco exposure (LfSmk), suggesting that compensatory mechanisms increase red blood cell count, hemoglobin, and hematocrit back to ‘normal’ in the context of chronic tobacco use.

Two aspects of these results were surprising. First, the effects of smoking were confined to erythroid traits. This contrasted the effects of obesity and adipose distribution, which impacted blood traits across lineages [26]. While we previously inferred the effects of BMI and WHR to impact hematopoietic stem and progenitor cell biology, the effects of tobacco and alcohol use seem mostly related to terminal erythroid maturation and/or erythrocyte homeostasis. While related genetic mechanisms require further exploration in functional studies, we anticipate the effects of tobacco and alcohol use impact erythrocyte stability, splenic clearance, or renal function (e.g., erythropoietin production).

Second, the negative effects of SmkInit on erythroid traits countered prevailing clinical intuition and prior observations linking tobacco use with higher hemoglobin levels and/or reduced anemia risk [9,12]. We note that an increased genetic predisposition to initiate smoking, as defined by GWAS [18], does not necessarily equate to long term tobacco use. While our experiments using LfSmk IVs argued against a direct positive effect of chronic smoking on hemoglobin levels, these findings may not completely capture the physiologic context of the long-term smokers who are generally profiled in clinical observational studies. Nutritional status, chronic disease, or other genetic predispositions like alcohol use may be unmeasured confounding variables in previous observational studies that reported effects of tobacco and alcohol use on red cell indices.

Blood cell formation and function impact myriad disease states, from heritable cytopenias to autoimmune disease and oncologic processes. In addition to tobacco and alcohol use behaviors, we anticipate that other systemic and environmental factors important for blood cell biology are not well characterized. A compilation of clinical observation, genetic evidence, and functional approaches will be necessary to reveal novel factors and mechanisms that impact blood cell formation and/or mature blood cell functions. Provided certain assumptions are met, MR can determine genetic trait interactions free from the bias and confounders inherent to observational studies [20]. Our results provide exemplary evidence that statistical genetics avenues can provide new perspectives on blood trait associations.

## Methods

### GWAS collection

We used publicly available GWAS summary statistics for the exposure traits related to alcohol and tobacco use [18,24], outcome variables related to quantitative blood measurements [16], and potential confounding and/or mediating traits (**S1 Table**). We focused on individuals of European descent due to sample size considerations (and consequently statistical power). All GWAS data used genome build hg19.

Traits related to tobacco use included lifetime smoking score (N = 462, 690 individuals)—a continuous trait calculated from smoking initiation, duration, and heaviness, based on UKBB data [24]; smoking initiation (SmkInit, N = 1,232,091)–a binary trait based on whether an individual had ever smoked regularly for >1 month or had more than 100 cigarettes (total) in one’s life [18]; smoking cessation (SmkCess, N = 547,219)–a binary trait based on whether an individual had at one point consistently used tobacco but subsequently quit [18]; and cigarettes smoked per day (CigPerDay, N = 337,334)–a continuous quantitative variable [18]. We also obtained GWAS summary statistics for SmkInit that excluded UKBB participants (https://conservancy.umn.edu) [30]. The only trait related to alcohol use was number of alcoholic drinks consumed per week (DrnkWk, N = 941,280) [18]. All alcohol and tobacco use behaviors were originally obtained via self-reporting, as described in the original GWAS. Quantitative blood traits included genetic associations of 15 phenotypes for red blood cell, white blood cell, and platelet indices (N = 563,085) [16]. Other variables considered as potential mediators or confounders included were body mass index (BMI) and waist-hip ratio (WHR) (N = 694,649 for both) [31], type 2 diabetes (T2D, N = 1,407,282) [32], coronary artery disease (CAD, N = 547.261) [33], depression (N = 829,616) [34], serum calcium (N = 332,355) [35], serum vitamin D levels (N = 346,130) [35], serum testosterone (N = 329,274) [35], and bone mineral density (BMD, N = 426,824) [36].

### Genetic variant selection and instrumental variable creation

We constructed genetic instrumental variables (IVs) by filtering summary GWAS statistics for SNPs common to exposure, outcome, and/or mediating factor data sets. We focused on European ancestry data, although in some cases included multi-ancestry data for mediating factors to optimize power. We used R v4.2.3 [37] and the TwoSampleMR package (v0.5.6, [38]) to clump SNPs meeting genome-wide significance for each exposure phenotype, selecting single SNPs in linkage disequilibrium (EUR r^2^<0.01) in 250 kb genomic regions. Instrumental variable strength was estimated using Cragg-Donald F-statistics. IVs calculated to have an F-statistic > 10 were deemed to have limited weak instrument bias [22].

### Mendelian randomization and mediation analyses

Certain assumptions must be true to permit valid conclusions from MR studies, including that independent genetic instruments (SNPs) must be associated with the exposure trait. Weak instruments (i.e., weak association with the risk factor and/or imprecise association with the outcome), horizontal pleiotropy (when genetic IVs affect the outcome through causal pathways unrelated to the exposure), heterogeneity in variant-specific effects, and errors in phenotype measurements (especially if these are biased) can limit applicability [20]. This study attempts to follow all best practices and reporting related to MR experiments. Given heterogeneity in some instrumental variables, we utilized random effects models.

We used the TwoSampleMR package (v0.5.6, [38]) to conduct two sample MR analyses. Two sample MR experiments focused on results from inverse variance weighted (IVW), weighted median (WM), and MR Egger regression methods. We looked for evidence of horizontal pleiotropy using MR Egger regression intercepts, as a significant deviation from zero can imply directional bias [39]. To estimate the independent direct effects of multiple potentially related exposures traits on the outcome variables, we ran multivariable MR (MVMR) using the MVMR package [21] and report IVW causal effect estimates. From MR Steiger analyses designed to infer correct directional relationships between two traits–i.e., to define which is exposure and which is outcome—we report sensitivity, statistical significance estimates, and inference for correct causal direction [40].

To assess the effect of mediator variables–i.e., those that are intermediate on a causal path starting at the IV for exposure and ending in the outcome—we conducted mediation experiments as previously described [25]. We report total and direct effects estimates for each exposure and mediating trait on given outcomes [41].

Our criterion for statistical significance was p<0.05, and 95% confidence intervals were calculated as 1.96 times the standard error. We ran all statistical analyses in R, and prepared figures using GraphPad Prism.

## Supporting information

S1 Fig Two sample MR effect estimates for genetically influenced SmkInit on the indicated blood traits. Effects of a 2-fold increase in SmkInit risk on the indicated blood traits by two sample MR. Bars indicate 95% confidence intervals. Trait abbreviations can be found in S1 Table. *p<0.05.
(PDF)

S2 Fig Sensitivity analyses for trait association in two sample MR experiments. (**A-C**) Effects of a 2-fold increase in SmkInit risk or a 1 SD unit increase in alcoholic drinks per week on (**A**) RBC, (**B**) HGB, or (**C**) HCT by weighted median (WM) and MR Egger regression analyses. IVW estimates also found in Fig 1 are presented here for comparison. Bars indicate 95% confidence intervals. Trait abbreviations can be found in S1 Table. *p<0.05.
(PDF)

S3 Fig Two sample MR effect estimates for a 1 SD unit increase in the indicated erythroid traits on SmkInit. None of the estimates reached statistical significance. Bars indicate 95% confidence intervals. Trait abbreviations can be found in S1 Table.
(PDF)

S4 Fig Two sample MR effect estimates for genetically influenced SmkInit (excluding UKBB participant data) on the indicated blood traits. Effects of a 2-fold increase in SmkInit risk (without UKBB participant data) on the indicated blood traits by two sample MR. Bars indicate 95% confidence intervals. Trait abbreviations can be found in S1 Table. *p<0.05.
(PDF)

S5 Fig Two sample MR effect estimates for a 1 SD unit increase in smoking traits on the indicated blood traits. Bars indicate 95% confidence intervals. Trait abbreviations can be found in S1 Table. *p<0.05.
(PDF)

S6 Fig Two sample MR experiments show effects of select cardiometabolic, cardiovascular, and psychiatric phenotypes on quantitative blood trait variation. We assessed the effects of instrumental variables for genetically influenced body mass index (BMI, 1244 SNPs) (Pulit et al., 2019) [31], waist-to-hip ratio (WHR, 621 SNPs) (Pulit et al., 2019) [31], Depression (58 SNPs) (Meng et al., 2024) [34], Type 2 Diabetes (T2D, 401 SNPs) (Vujkovic et al., 2020) [32], coronary artery disease (CAD, 208 SNPs) (Van Der Harst and Verweij, 2018) [33], serum Calcium level (225 SNPs) (Sinnott-Armstrong et al., 2021) [35], serum Vitamin D level (98 SNPs) (Sinnott-Armstrong et al., 2021) [35], Testosterone level (95 SNPs) (Sinnott-Armstrong et al., 2021) [35], and bone mineral density (BMD, 896 SNPs) (Morris et al., 2019) [36] for blood trait effects. Bars indicate 95% confidence intervals. Trait abbreviations can be found in S1 Table. *p<0.05.
(PDF)

S7 Fig MVMR experiments analyzing the effect of SmkInit on the indicated blood traits after adjusting for body mass index (BMI). Bars indicate 95% confidence intervals. Trait abbreviations can be found in S1 Table. *p<0.05.
(PDF)

S8 Fig Two sample MR effect estimates for a 1 SD unit increase in DrnkWk on the indicated blood traits. Bars indicate 95% confidence intervals. Trait abbreviations can be found in S1 Table. *p<0.05.
(PDF)

S9 Fig MVMR effect estimates for SmkInit or DrnkWk on the indicated blood traits. All experiments used an instrumental variable for SmkInit adjusted for DrnkWk in an MVMR experiment. The effects of SmkInit and DrnkWk are shown. After adjustment, SmkInit did not have significant effects on any blood trait whereas DrnkWk did retain some significant effects. Bars indicate 95% confidence intervals. Trait abbreviations can be found in S1 Table. *p<0.05.
(PDF)

S10 Fig MVMR effect estimates for LfSmk or DrnkWk on the indicated blood traits. All experiments used an instrumental variable for LfSmk adjusted for DrnkWk in an MVMR experiment. The effects of LfSmk and DrnkWk are shown. After adjustment, LfSmk only retained significant effects on WBC, and NEU, RDW. Bars indicate 95% confidence intervals. *p<0.05.
(PDF)

S1 Table Explanations of genetically influenced traits analyzed in our study, including GWAS sources (PMID) and GWAS sample sizes. (XLSX)

S2 Table Craig-Donald F statistics for instrumental variables used in this study. (XLSX)
