# Supplementary material for: Genetically influenced tobacco and alcohol use behaviors impact erythroid trait variation
Source: PLoS One. 2024 Sep 5;19(9):e0309608. doi: 10.1371/journal.pone.0309608 (PMC11376579; doi:10.1371/journal.pone.0309608)
Supplement: S1 Fig — Effects of a 2-fold increase in SmkInit risk on the indicated blood traits by two sample MR. Bars indicate 95% confidence intervals. Trait abbreviations can be found in S1 Table. *p<0.05. (PDF) [file pone.0309608.s001.pdf]

Supplementary Information

Genetically influenced tobacco and alcohol use behaviors impact erythroid trait variation

Shriya Shivakumar[1,2], Madison B Wilken[1], Victor Tsao[1], Bárbara D. Bitarello[2], Christopher S Thom[1,3,*]

[1]Division of Neonatology, Children's Hospital of Philadelphia, Philadelphia, PA, USA
[2]Biology Department, Bryn Mawr College, Bryn Mawr, PA, USA
[3]Department of Pediatrics, University of Pennsylvania Perelman School of Medicine, Philadelphia, PA, USA

*Correspondence:
Christopher S Thom, MD, PhD
Children's Hospital of Philadelphia
10-052 Colket Translational Research Bldg
3501 Civic Center Blvd
Philadelphia, PA 19104
thomc@chop.edu

Supplementary Figures

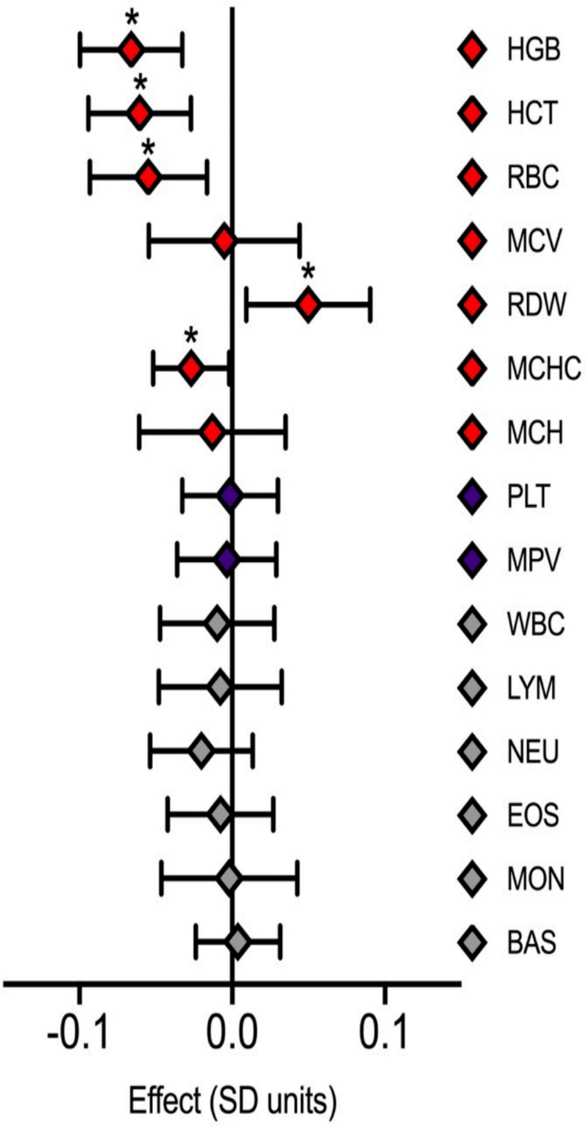

Supplementary Figure 1. Two sample MR effect estimates for genetically influenced SmkInit on the indicated blood traits. Effects of a 2-fold increase in SmkInit risk on the indicated blood traits by two sample MR. Bars indicate 95% confidence intervals. Trait abbreviations can be found in Supplementary Table 1. *$p<0.05$.
