# Supplementary material for: Genetically influenced tobacco and alcohol use behaviors impact erythroid trait variation
Source: PLoS One. 2024 Sep 5;19(9):e0309608. doi: 10.1371/journal.pone.0309608 (PMC11376579; doi:10.1371/journal.pone.0309608)
Supplement: S2 Fig — (A-C) Effects of a 2-fold increase in SmkInit risk or a 1 SD unit increase in alcoholic drinks per week on (A) RBC, (B) HGB, or (C) HCT by weighted median (WM) and MR Egger regression analyses. IVW estimates also found in Fig 1 are presented here for comparison. Bars indicate 95% confidence intervals. Trait abbreviations can be found in S1 Table. *p<0.05. (PDF) [file pone.0309608.s002.pdf]

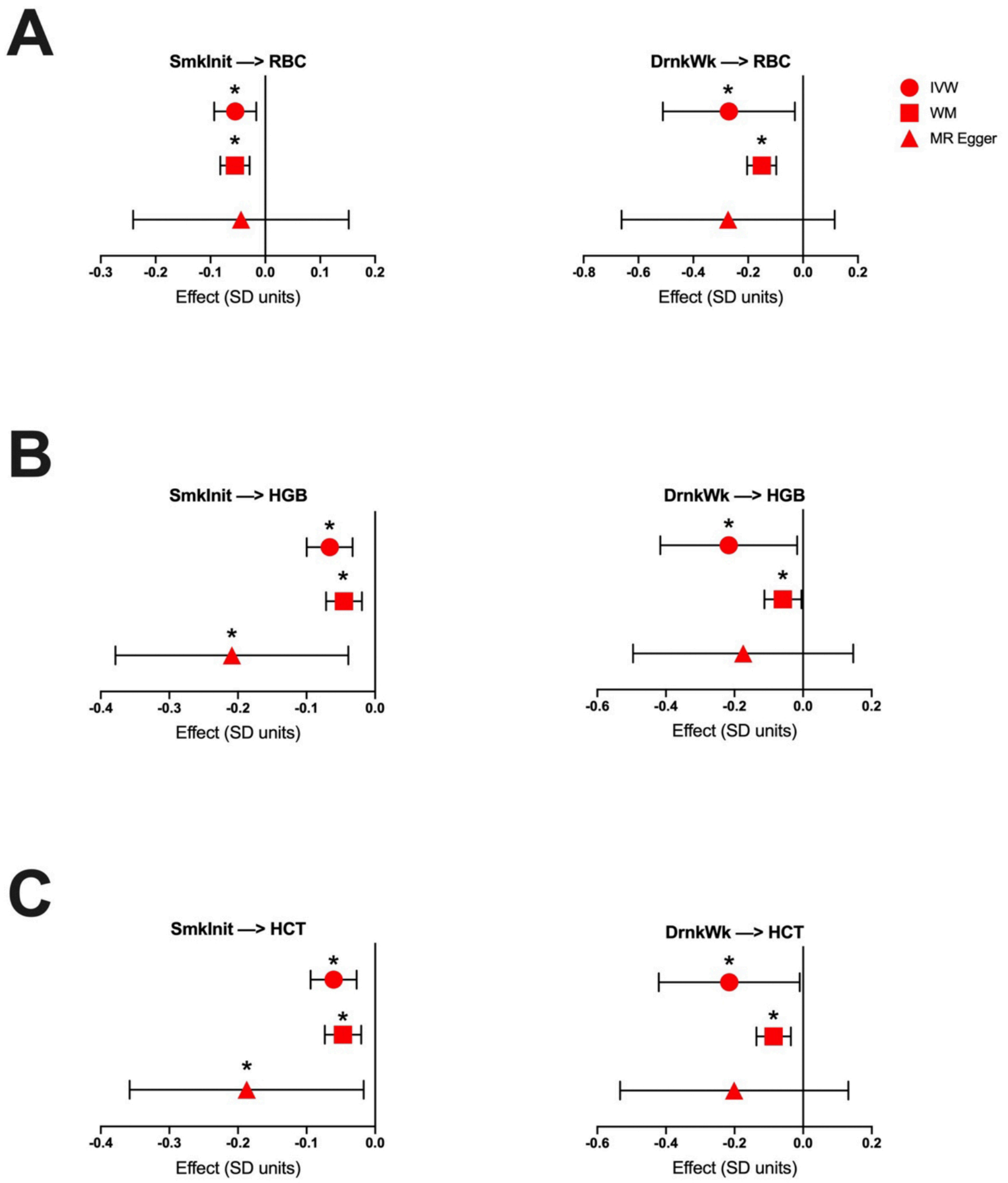

Supplementary Figure 2. Sensitivity analyses for trait association in two sample MR experiments. (**A-C**) Effects of a 2-fold increase in SmkInit risk or a 1 SD unit increase in alcoholic drinks per week on (**A**) RBC, (**B**) HGB, or (**C**) HCT by weighted median (WM) and MR Egger regression analyses. IVW estimates also found in Figure 1 are presented here for comparison. Bars indicate 95% confidence intervals. Trait abbreviations can be found in Supplementary Table 1. *p<0.05.
