# Supplementary material for: Genetically influenced tobacco and alcohol use behaviors impact erythroid trait variation
Source: PLoS One. 2024 Sep 5;19(9):e0309608. doi: 10.1371/journal.pone.0309608 (PMC11376579; doi:10.1371/journal.pone.0309608)
Supplement: S3 Fig — None of the estimates reached statistical significance. Bars indicate 95% confidence intervals. Trait abbreviations can be found in S1 Table. (PDF) [file pone.0309608.s003.pdf]

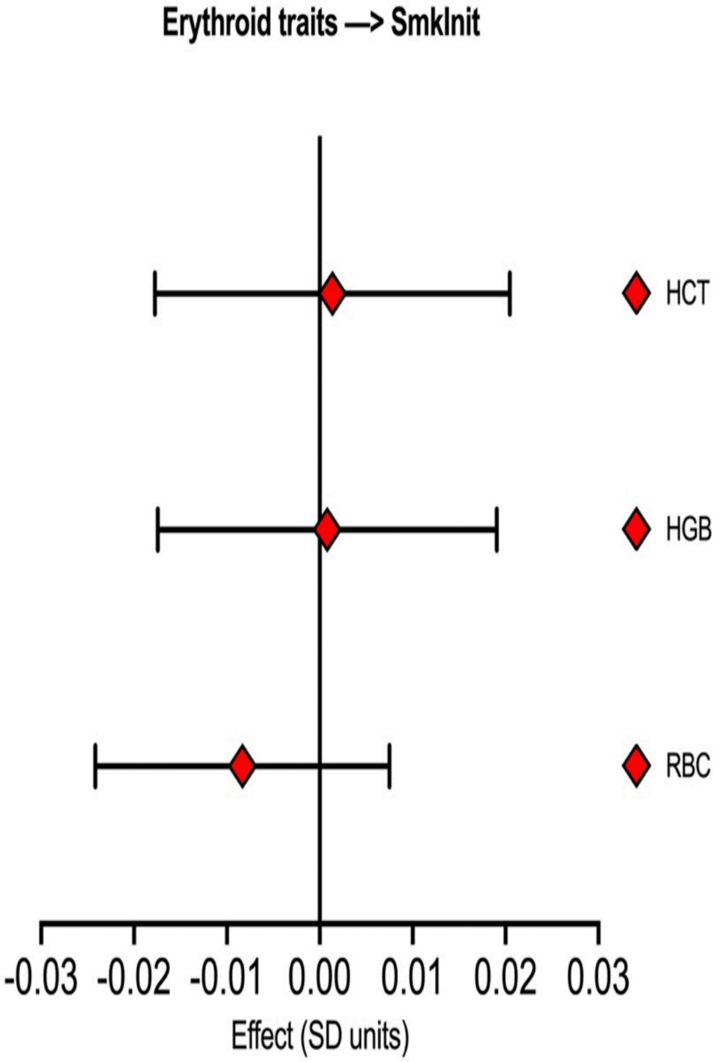

Supplementary Figure 3. Two sample MR effect estimates for a 1 SD unit increase in the indicated erythroid traits on SmkInit. None of the estimates reached statistical significance. Bars indicate 95% confidence intervals. Trait abbreviations can be found in Supplementary Table 1.
