# Supplementary material for: Genetically influenced tobacco and alcohol use behaviors impact erythroid trait variation
Source: PLoS One. 2024 Sep 5;19(9):e0309608. doi: 10.1371/journal.pone.0309608 (PMC11376579; doi:10.1371/journal.pone.0309608)
Supplement: S6 Fig — We assessed the effects of instrumental variables for genetically influenced body mass index (BMI, 1244 SNPs) (Pulit et al., 2019) [31], waist-to-hip ratio (WHR, 621 SNPs) (Pulit et al., 2019) [31], Depression (58 SNPs) (Meng et al., 2024) [34], Type 2 Diabetes (T2D, 401 SNPs) (Vujkovic et al., 2020) [32], coronary artery disease (CAD, 208 SNPs) (Van Der Harst and Verweij, 2018) [33], serum Calcium level (225 SNPs) (Sinnott-Armstrong et al., 2021) [35], serum Vitamin D level (98 SNPs) (Sinnott-Armstrong et al., 2021) [35], Testosterone level (95 SNPs) (Sinnott-Armstrong et al., 2021) [35], and bone mineral density (BMD, 896 SNPs) (Morris et al., 2019) [36] for blood trait effects. Bars indicate 95% confidence intervals. Trait abbreviations can be found in S1 Table. *p<0.05. (PDF) [file pone.0309608.s006.pdf]

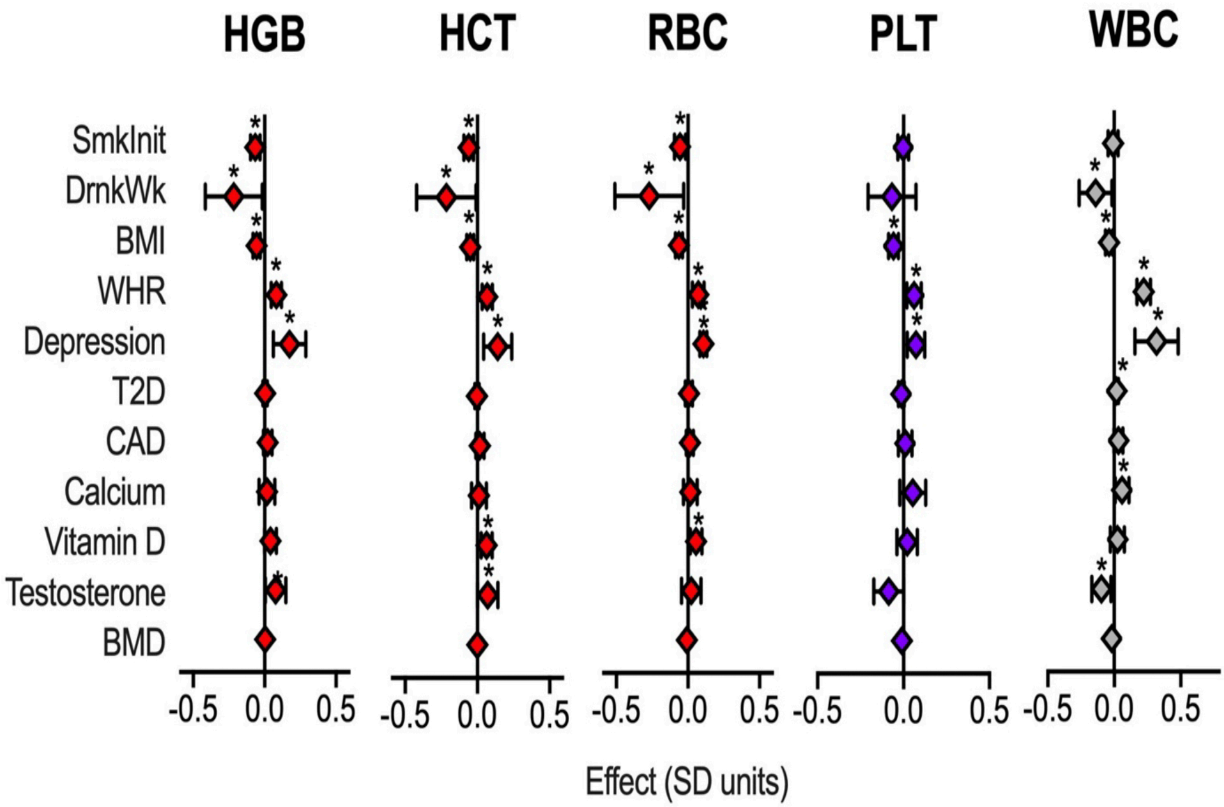

Supplementary Figure 6. Two sample MR experiments show effects of select cardiometabolic, cardiovascular, and psychiatric phenotypes on quantitative blood trait variation. We assessed the effects of instrumental variables for genetically influenced body mass index (BMI, 1244 SNPs) (Pulit et al., 2019), waist-to-hip ratio (WHR, 621 SNPs) (Pulit et al., 2019), Depression (58 SNPs) (Meng et al., 2024), Type 2 Diabetes (T2D, 401 SNPs) (Vujkovic et al., 2020), coronary artery disease (CAD, 208 SNPs) (Van Der Harst and Verweij, 2018), serum Calcium level (225 SNPs) (Sinnott-Armstrong et al., 2021), serum Vitamin D level (98 SNPs) (Sinnott-Armstrong et al., 2021), Testosterone level (95 SNPs) (Sinnott-Armstrong et al., 2021), and bone mineral density (BMD, 896 SNPs) (Morris et al., 2019) for blood trait effects. Bars indicate 95% confidence intervals. Trait abbreviations can be found in Supplementary Table 1. *$p<0.05$.
