# Supplementary material for: Genetically influenced tobacco and alcohol use behaviors impact erythroid trait variation
Source: PLoS One. 2024 Sep 5;19(9):e0309608. doi: 10.1371/journal.pone.0309608 (PMC11376579; doi:10.1371/journal.pone.0309608)
Supplement: S8 Fig — Bars indicate 95% confidence intervals. Trait abbreviations can be found in S1 Table. *p<0.05. (PDF) [file pone.0309608.s008.pdf]

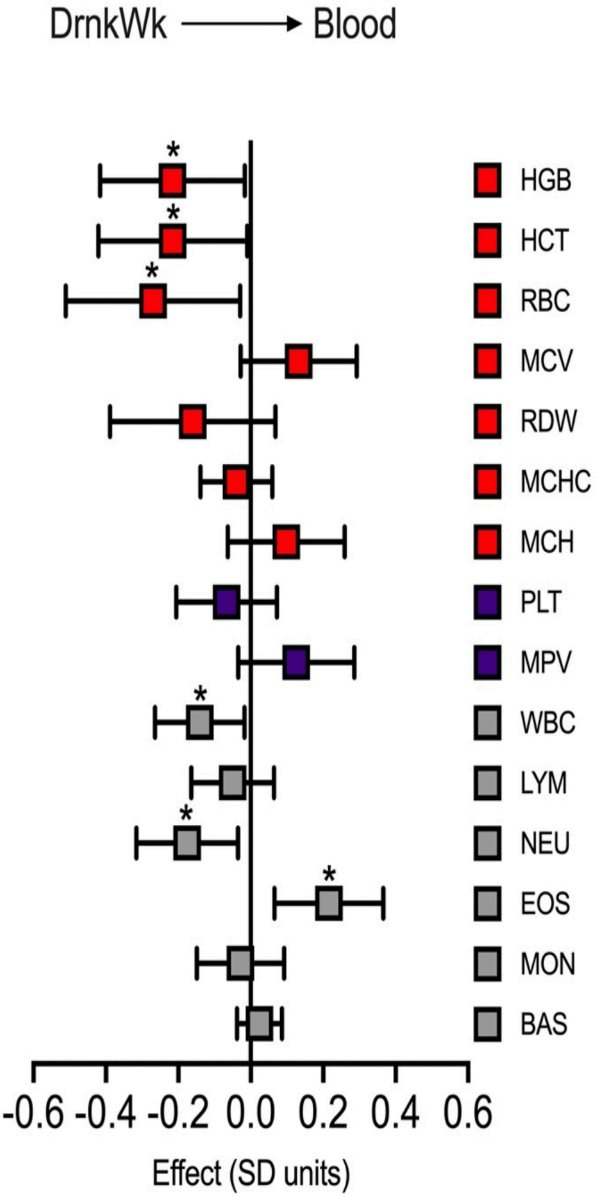

Supplementary Figure 8. Two sample MR effect estimates for a 1 SD unit increase in DrnkWk on the indicated blood traits. Bars indicate 95% confidence intervals. Trait abbreviations can be found in Supplementary Table 1. *p<0.05.
