# Supplementary material for: Genetically influenced tobacco and alcohol use behaviors impact erythroid trait variation
Source: PLoS One. 2024 Sep 5;19(9):e0309608. doi: 10.1371/journal.pone.0309608 (PMC11376579; doi:10.1371/journal.pone.0309608)
Supplement: S9 Fig — All experiments used an instrumental variable for SmkInit adjusted for DrnkWk in an MVMR experiment. The effects of SmkInit and DrnkWk are shown. After adjustment, SmkInit did not have significant effects on any blood trait whereas DrnkWk did retain some significant effects. Bars indicate 95% confidence intervals. Trait abbreviations can be found in S1 Table. *p<0.05. (PDF) [file pone.0309608.s009.pdf]

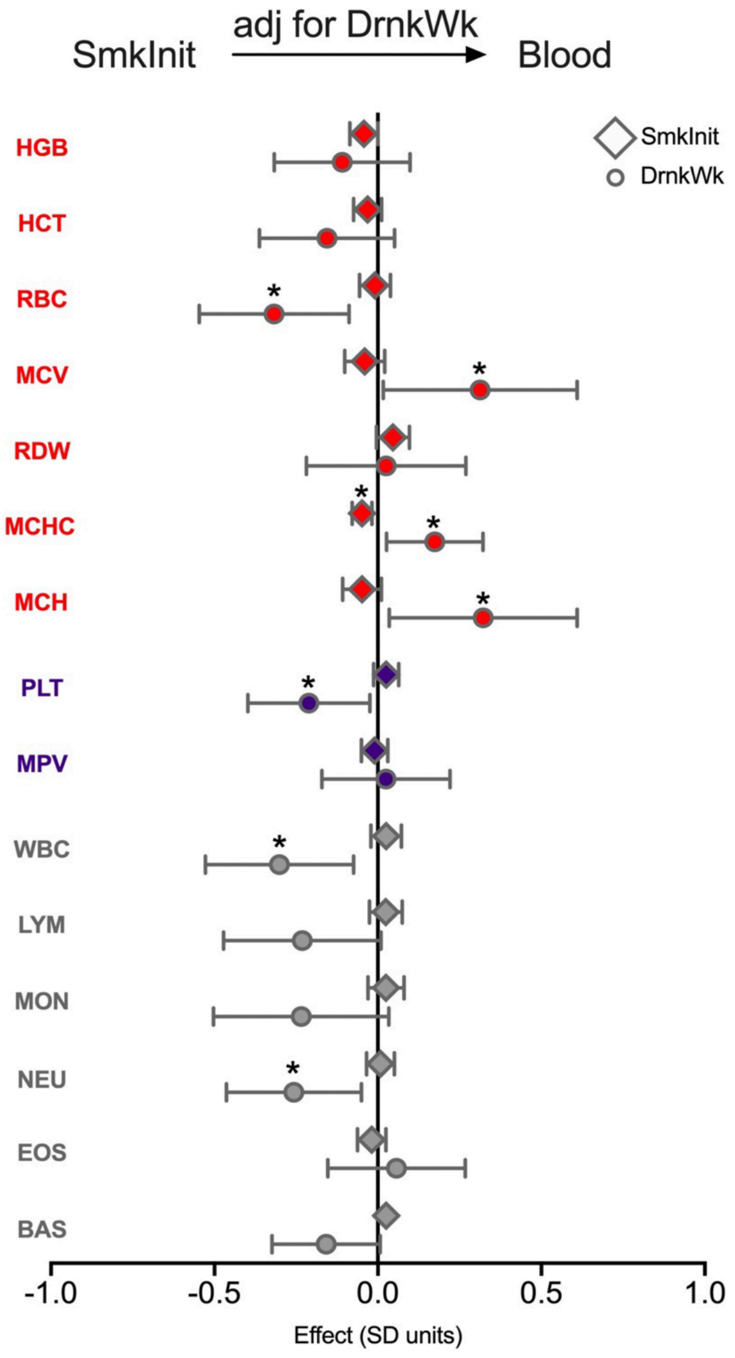

Supplementary Figure 9. MVMR effect estimates for SmkInit or DrnkWk on the indicated blood traits. All experiments used an instrumental variable for SmkInit adjusted for DrnkWk in an MVMR experiment. The effects of SmkInit and DrnkWk are shown. After adjustment, SmkInit did not have significant effects on any blood trait whereas DrnkWk did retain some significant effects. Bars indicate 95% confidence intervals. Trait abbreviations can be found in Supplementary Table 1. *$p<0.05$.
