# Supplementary material for: Genetically influenced tobacco and alcohol use behaviors impact erythroid trait variation
Source: PLoS One. 2024 Sep 5;19(9):e0309608. doi: 10.1371/journal.pone.0309608 (PMC11376579; doi:10.1371/journal.pone.0309608)
Supplement: S10 Fig — All experiments used an instrumental variable for LfSmk adjusted for DrnkWk in an MVMR experiment. The effects of LfSmk and DrnkWk are shown. After adjustment, LfSmk only retained significant effects on WBC, and NEU, RDW. Bars indicate 95% confidence intervals. *p<0.05. (PDF) [file pone.0309608.s010.pdf]

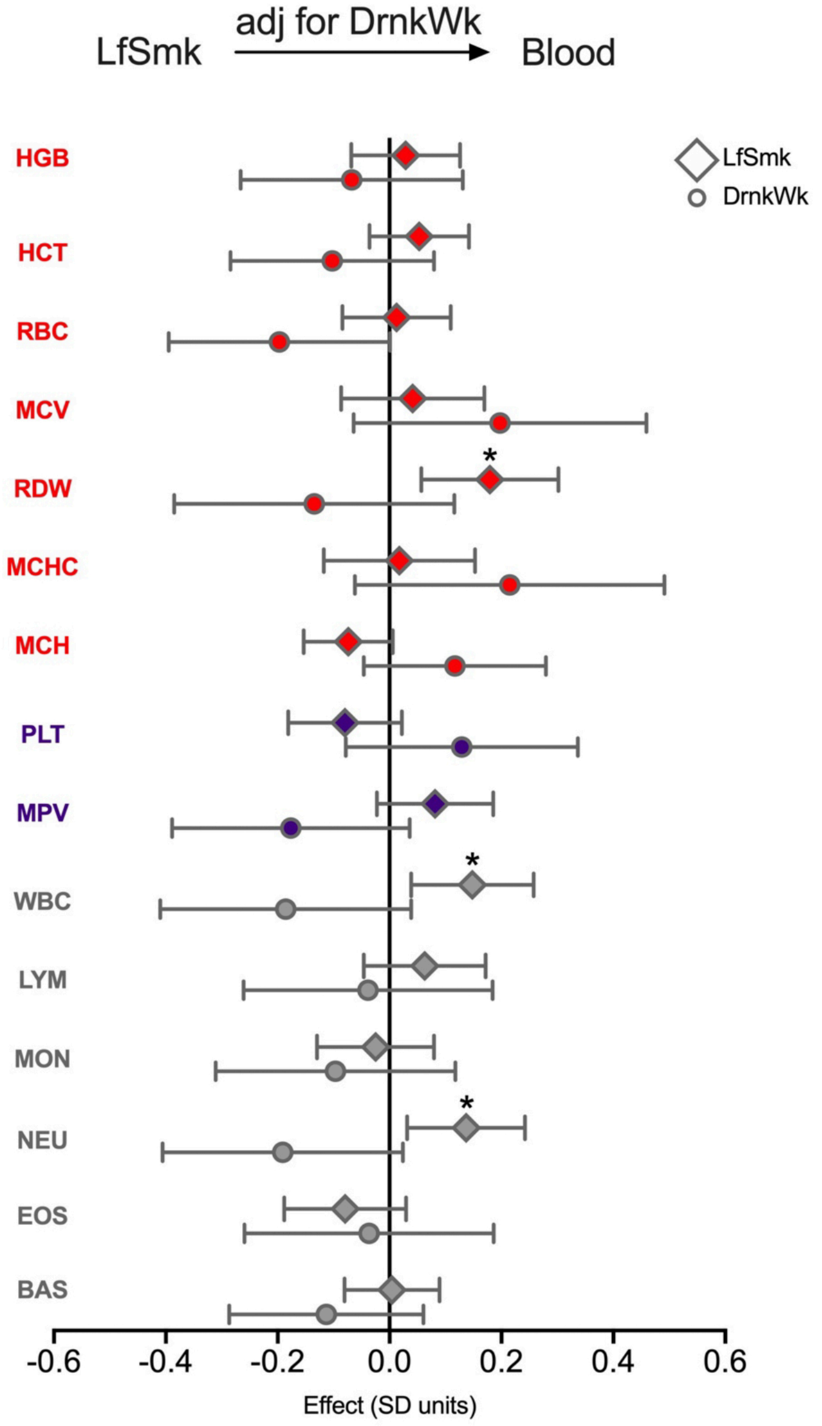

Supplementary Figure 10. MVMR effect estimates for LfSmk or DrnkWk on the indicated blood traits. All experiments used an instrumental variable for LfSmk adjusted for DrnkWk in an MVMR experiment. The effects of LfSmk and DrnkWk are shown. After adjustment, LfSmk only retained significant effects on WBC, and NEU, RDW. Bars indicate 95% confidence intervals. *p<0.05.
